# Supplementary material for: Controllability over stressor decreases responses in key threat-related brain areas
Source: Commun Biol. 2021 Jan 5;4:42. doi: 10.1038/s42003-020-01537-5 (PMC7785729; doi:10.1038/s42003-020-01537-5)
Supplement: Supplementary file 2 — Supplementary materials [file 42003_2020_1537_MOESM2_ESM.pdf]

Supplementary materials

Controllability over stressor decreases responses in key threat-related brain areas

Chirag Limbachia, Kelly Morrow, Anastasiia Khibovska, Christian Meyer, Srikanth Padmala, and Luiz Pessoa

Communications Biology

Corresponding author: Luiz Pessoa (pessoa@umd.edu)

Supplementary Methods

Anxiety questionnaires

Participants completed the trait portion of the Spielberger State-Trait Anxiety Inventory STAI[1]; before scanning, and then completed the state portion immediately before the scanning session. The STAI scale is defined such that scores range from 20 to 80. In a recent analysis of over 3,000 individuals across behavioral and fMRI studies, the mode was a trait score of 36, and only 5% of the participants exhibited scores above 60[2]. Given our participant-matching procedure (see next), participants with trait anxiety scores above 60 were removed from the study.

Participant matching

Participants completed the trait portion of the STAI during an initial screening interview. The earliest recruited participants were assigned to the controllable condition of the experiment. Each participant's trait anxiety score and biological sex were used to find a match for the uncontrollable condition. In matching participants, we attempted to keep the difference in trait scores to within +/- 4 points. We prioritized matching new participants with already scanned participants. However, if we could not find a match with a +/- 4-point difference, we matched a new participant with participants who were already screened but not yet scanned. In such cases, we randomly assigned a participant to the controllable or uncontrollable condition. When necessary, matching of a new participant had to wait until a new one was screened who had an anxiety score within +/4 points. Overall, the time to recruit a match for controllable participants varied from one day up to one month.

MRI data acquisition

Functional and structural MRI data were acquired using a 3T Siemens TRIO scanner with a 32-channel head coil. First, a high-resolution T2-weighted anatomical scan using Siemens's SPACE sequence (0.8 mm isotropic) was collected. Subsequently, we collected functional EPI volumes using a multiband scanning sequence[3] with TR = 1.0 sec, TE = 39 ms, FOV = 210 mm, and multiband factor = 6. Each volume contained 66 non-overlapping oblique slices oriented 30° clockwise relative to the AC-PC axis (2.2 mm isotropic). A high-resolution T1-weighted MPRAGE anatomical scan (0.8 mm isotropic) was collected. Additionally, in each session, double-echo field maps (TE1 = 4.92 ms, TE2 = 7.38 ms) were acquired with acquisition parameters matched to the functional data.

Functional MRI preprocessing

We adopted the same procedures used in our previous study to minimize the impact of image distortion and improve spatial localization[4], see also[5]. To preprocess the functional and anatomical MRI data, we used a combination of packages and in-house scripts. The first three volumes of each functional run were discarded to account for equilibration effects. Slice-timing correction used the Analysis of Functional Neuroimages' AFNI;[6] 3dTshift with Fourier interpolation to align the onset times of every slice in a volume to the first acquisition slice. To reduce the contribution of head motion, we employed FSL's Independent Component Analysis, Automatic Removal of Motion Artifacts (ICA-AROMA)[7]. Components classified as head motion are regressed out of the functional MRI data with FSL's fsl_regfilt.

In this study, we strived to improve functional-to-anatomical co-registration given the small size of some of the structures of interest. Skull stripping determines which voxels are considered part of the brain and plays an important role in successful subsequent co-registration and normalization steps. Currently, available packages perform sub-optimally in specific cases, and mistakes in the brain-to-skull

segmentation can be easily identified. Accordingly, to skull strip the T1 high-resolution anatomical image (which was rotated to match the oblique plane of the functional data with AFNI's 3dWarp), we employed six different packages [ANTs[8]: http://stnava.github.io/ANTs/; AFNI[6]: http://afni.nimh.nih.gov/; ROBEX[9]: https://www.nitrc.org/projects/robex; FSL[5]: http://fsl.fmrib.ox.ac.uk/fsl/fslwiki/; SPM: http://www.fil.ion.ucl.ac.uk/spm/; and BrainSuite[10]: http://brainsuite.org/] and employed a "voting scheme" as follows[4,5]: based on T1 data, a voxel was considered to be part of the brain if 4/6 packages estimated it to be a brain voxel; otherwise the voxel was not considered to be brain tissue.

Subsequently, FSL was used to process field map images and create a phase-distortion map for each participant (by using bet and fsl_prepare_fieldmap). FSL's epi_reg was then used to apply boundary-based co-registration to align the unwarped mean volume registered EPI image with the skull-stripped anatomical image (T1 or T2), along with simultaneous EPI distortion-correction[11].

Next, ANTS was used to estimate a nonlinear transformation that mapped the skull-stripped anatomical image (T1 or T2) to the skull-stripped MNI152 template (interpolated to 1-mm isotropic voxels). Finally, ANTS combined the nonlinear transformations from co-registration/unwarping (from mapping mean functional EPI image to the anatomical T1 or T2) and normalization (from mapping T1 or T2 to the MNI template) into a single transformation that was applied to map volume-registered functional volumes to standard space (interpolated to 2-mm isotropic voxels). In this process, ANTS also utilized the field maps to simultaneously minimize EPI distortion.

Voxelwise analysis

The voxelwise analysis followed the same model described in equation (1). Thus, the analyses were the same, with the exception of the multilevel component (ROIs). The cluster extent for statistical thresholding was determined by simulations using the 3dClustSim program and other AFNI tools. For

these simulations, the smoothness of the data was estimated using 3dFWHMx (restricted to gray matter voxels) based on the residual time series from the participant-level voxelwise analysis. Taking into account the recent report of increased false-positive rates linked to the assumption of Gaussian spatial autocorrelation in fMRI data[12], we used the –acf (i.e., autocorrelation function) option added to the 3dFWHMx and 3dClustSim tools, which models spatial noise as a mixture of Gaussian plus monoexponential distributions. This improvement was shown to control false-positive rates around the desired alpha level, especially with relatively stringent voxel-level uncorrected p values such as 0.001[13]. Based on a voxel-level uncorrected p value of 0.001, simulations indicated a minimum cluster extent of 13 voxels for a cluster-level corrected alpha of 0.05.

Visualization of response shape

Estimation of stressor responses relied on simultaneously accounting for all signal contributions, including fMRI responses during approach and retreat periods surrounding the stressor. To do so, response magnitude estimation of stressor events relied on convolving regressors with a canonical hemodynamic response. In doing so, regression coefficients are efficiently estimated for all conditions, allowing us to estimate responses to stressors. But to aid visualization of the stressor response, we performed an additional unassumed-shape analysis (also called deconvolution analysis) that estimated signal intensities at every time point following stressor delivery for a window of 13.75 seconds. We stress that the objective of this analysis was to help visualize the responses, and not to draw statistical inferences.

Responses were recovered using the 3dDeconvolve AFNI program using cubic spline basis functions, which provide a slightly smoother approximation of the underlying signal than using delta ("stick") functions (also called finite impulse responses or FIR). The results (Figures 4 and 7) confirm that the canonical hemodynamic response provided an adequate response model for most brain regions,

including the anticipated peak around 5 seconds post onset. However, deconvolution indicated that the canonical model was probably not ideal for some regions, such as the anterior hippocampus, PCC, and PCC/precuneus, which appeared to follow a different time evolution (note that the mismatch is not because the response was negative, but related to the shape/timing of the response itself).

Finally, we note that we chose to perform the main inferential analyses (described in the preceding sections) by using regressors convolved with the hemodynamic response because we did not have a priori information concerning the timing/shape of stressor-related responses. Importantly, stressor duration varied to some extent for each event, which poses a problem for deconvolution (essentially, each trial's response varied because of timing differences and potentially due to "noise" fluctuations).

Skin conductance response (SCR) analysis

For the analysis of SCR, we employed methods previously adopted in our past work e.g.,[4]. Each participant's SCR data were initially smoothed with a median-filter over 50 samples (200 ms) to reduce scanner-induced noise. Previous work has capitalized on the slow evolution of SCR signals to propose an analysis approach paralleling the one used with fMRI (Bach et al.[14] and Engelmann et al.[15]). Accordingly, the pre-processed SCR data were analyzed using multiple linear regression using the 3dDeconvolve program in AFNI. We employed the same regression model as the one used for fMRI data. All regressors were convolved with a canonical skin conductance response model based on the sigmoid-exponential function[16].

Relationship between SCR and brain activity: Trial-by-trial analysis

To probe the relationship between brain activity and physiological arousal, we focused on the BST and the left dorsal anterior insula (see Results). Our goal was to evaluate the relationship between trial-by-trial

responses in the brain and SCR. To estimate single-trial responses of fMRI data, the model was the same one employed for the participant-level analysis, except for the stressor regressor, which was defined separately for each stressor event ("trial"). Because the simultaneous presence of all trial-level stressor regressors in the model would unduly increase collinearity, estimation was performed via the iterative procedure developed by Mumford et al. [17] as implemented by the 3dLSS program of the AFNI suite.

The same approach was employed to estimate trial-by-trial SCRs. General methods for SCR processing followed our past work e.g.,[4]. For computational tractability, SCR data were resampled by decimating the original 250 Hz sample rate to 10 Hz. Note that given the slow evolution of this type of signal, the downsampled time series preserved the information needed for the analysis. As with fMRI data, 3dLSS was employed to estimate single-trial responses.

Bayesian multilevel model

The basic linear model can be written as

$$y_i = \alpha + \beta x_i + \varepsilon$$

where x is a predictor variable. A multilevel model with a grouping/clustering variable (for example, ROIs) indexed by j, can be expresses as

$$y_i = \alpha + \alpha_{j[i]} + \beta x_i + \beta_{j[i]} x_i + \varepsilon$$

where α is the overall intercept, β is the overall slope, $\alpha_{j[i]}$ are j intercepts, and $\beta_{j[i]}$ are j slopes (say, one per ROI). With two regressors, we can write

$$y_i = \alpha + \alpha_{j[i]} + \beta_{1,j[i]} x_{1,i} + \beta_{2,j[i]} x_{2,i} + \varepsilon.$$

In the present study, the difference of stressor response, $\Delta_{p,r}$, for a yoked pair of participants p and ROI r can be expressed in terms of the linear mixed effects model notation (lme4-like) as

$$\Delta_{p,r} \sim 1 + (1 \mid \text{PP}) + \text{StateMean} + \text{StateDiff} + \text{TraitMean} + \text{TraitDiff} + \text{ButtonDiff}$$
$$+ (1 + \text{StateMean} + \text{StateDiff} + \text{TraitMean} + \text{TraitDiff} + \text{ButtonDiff} \mid \text{ROI})$$

where PP (participant pair) and ROI are grouping variables, and the other terms indicate the covariates in the model. The first line specifies the intercepts, with a general one, as well as one per participant pair; it also specifies the 5 covariates (slopes). The second line specifies slopes for the covariates nested within ROI. The notation "1 + covariate" indicates that they are allowed to have a nonzero correlation with the intercepts (technically specified via a variance/covariance matrix).

The full model is therefore in the notation of [18]:

$$\Delta_{p,r} \sim \text{Student t}(\nu, \mu, \sigma)$$

$$\mu_{p,r} = \alpha + \alpha_{\text{PP}[p]} + \alpha_{\text{ROI}[r]} + \quad (1)$$
$$\beta_{\text{StateMean}}\text{StateMean} + \beta_{\text{StateDiff}}\text{StateDiff} + \quad (2)$$
$$\beta_{\text{TraitMean}}\text{TraitMean} + \beta_{\text{TraitDiff}}\text{TraitDiff} + \quad (3)$$
$$\beta_{\text{ButtonPressDiff}}\text{ButtonPressDiff} + \quad (4)$$
$$\gamma_{\text{StateMean},[r]}\text{StateMean} + \gamma_{\text{StateDiff},[r]}\text{StateDiff} + \quad (5)$$
$$\gamma_{\text{TraitMean},[r]}\text{TraitMean} + \gamma_{\text{TraitDiff},[r]}\text{TraitDiff} + \quad (6)$$
$$\gamma_{\text{TraitDiff},[r]}\text{ButtonPressDiff}$$
$$+ \epsilon \quad (7)$$

where the α terms are "intercepts", with the "varying intercepts" α_p and α_r capturing the contribution of each participant pair p and each ROI r. The slope parameters β are parameters that model the contributions of the covariates, and the "varying slopes", γ_r, model the covariate contribution at each specific ROI.

In addition, the following weakly-informative priors were employed. All intercept and slopes were defined as

$$\alpha \sim Student\_t(3,0,10)$$

$$\beta \sim Student\_t(3,0,10)$$

for all five covariates. The notation for the Student t distribution, $Student\_t(\nu, \mu, \sigma)$, is such that ν is the degrees of freedom, μ is the mean, and σ is the scale parameter. This prior is a very noninformative heavy-tail distribution suggested by Stan.

The intercepts clustered by participant were defined as

$$\alpha_{\mathrm{PP}} \sim Student\_t(\nu_{\mathrm{PP}}, 0, \sigma_{\mathrm{PP}})$$

$$\nu_{\mathrm{PP}} \sim Gamma(3.325{,}0.1)$$

$$\sigma_{\mathrm{PP}} \sim HalfStudent\ t(3{,}0{,}10)$$

where the Gamma distribution, $Gamma(k, \beta)$, is such that k is the shape parameter and β is the rate parameter. Note that α_{PP} expresses a vector of intercept parameters, one for each participant pair. The default suggested by Stan is $Gamma(2{,}0.1)$. However, we chose our parameters because they provide 50% probability mass that a value can be above/below 30. As the Gamma prior informs the estimate of the degrees of freedom, a value of $\nu > 30$ essentially entails a normal distribution. Values less than 30 will amount to heavier tails and more skew/outlier tolerance. In any case, the actual estimates of ν are estimated from the data, and the Gamma prior is rather weakly informative. The $HalfStudent\_t(\nu, \mu, \sigma)$ is simply a positively truncated Student t.

In a similar fashion, the slopes clustered by ROI were defined as

$$\gamma_{\mathrm{ROI}} \sim Student\_t(\nu_{\mathrm{ROI}}, 0, \sigma_{\mathrm{ROI}})$$

$$\nu_{\mathrm{ROI}} \sim Gamma(3.325{,}0.1)$$

$$\sigma_{\mathrm{ROI}} \sim HalfStudent\ t(3{,}0{,}10).$$

Finally, for the variance-covariance structure, the LKJ correlation prior[19] was used with the shape parameter with the value 1.

Supplementary Figures

Supplementary figure 1: Covariate posterior distributions

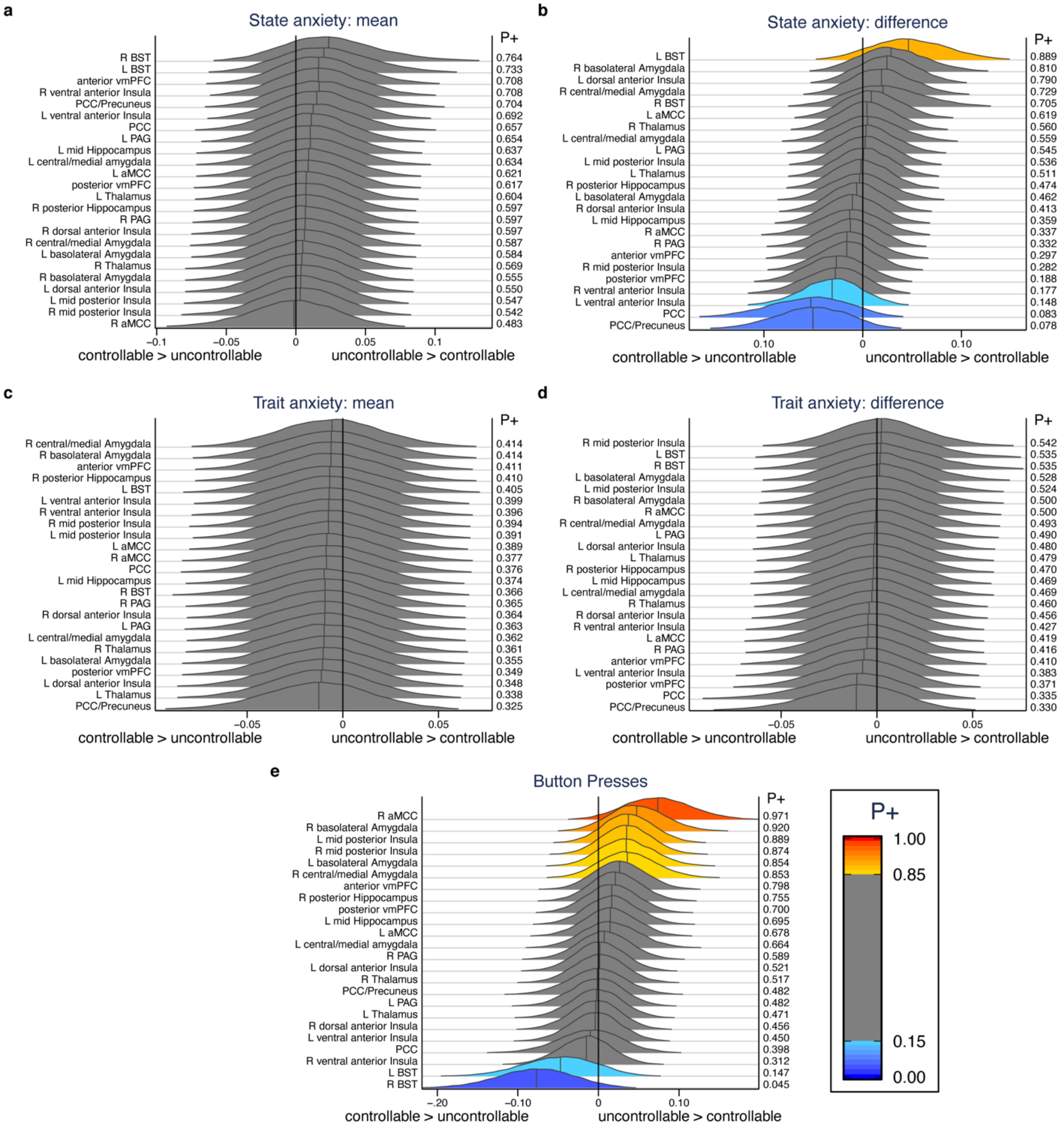

Evidence for individual differences in state/trait anxiety (**a-d**) and button presses (**e**). **a)** There is no relationship between yoked participants mean state anxiety score and the stressor contrast (uncontrollable – controllable) for any of the ROIs. **b)** There is some evidence suggesting that state anxiety difference (uncontrollable – controllable) has a positive relationship with the stressor contrast for left BST, such that yoked participants with greater state anxiety difference exhibit larger stressor contrast. There is also

evidence suggesting that state anxiety difference has a negative relationship with the stressor contrast for regions like PCC/Precuneus and left ventral anterior insula, such that yoked participants with greater state anxiety difference exhibit smaller stressor contrast. The **c)** mean trait score of the yoked participants or its **d)** difference does not have any relationship with stressor contrast. **e)** There is evidence suggesting that difference in the total number of button presses between the yoked participants has positive relationship with the stressor contrast for regions like right aMCC, right basolateral amygdala, left mid-posterior Insula, right mid-posterior Insula, left basolateral amygdala, and left central/medial amygdala; and negative relationship with the stressor contrast for regions like left and right BST. Abbreviations: aMCC, anterior midcingulate cortex; BST, bed nucleus of the stria terminalis; PCC, posterior cingulate cortex.

Supplementary figure 2: Activation clusters with controllability effect

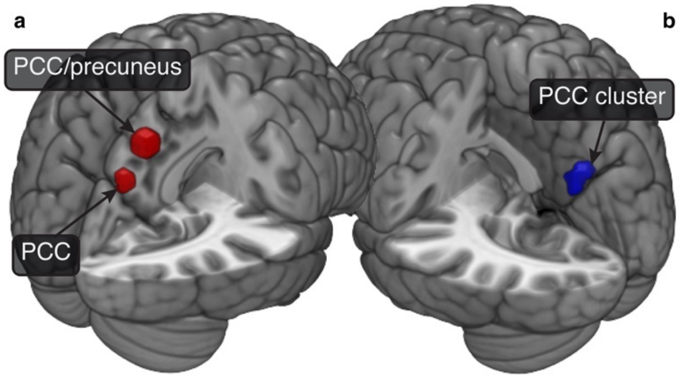

a PCC/precuneus and PCC regions of interest and **b** activation cluster with a controllability effect from voxelwise analysis. Abbreviations: PCC, posterior cingulate cortex.

Supplementary Tables

Supplementary Table 1: Uncontrollable stressor vs. Controllable stressor

Hemisphere	Area	MNI coordinates	Cluster extent (voxels)	t-value at peak coordinate
Uncontrollable > Controllable				
L	Middle/inferior occipital gyrus	-46 -84 -2	17	4.100
R	Middle occipital gyrus	30 -78 16	24	4.523
R	Fusiform gyrus (1)	28 -74 -12	14	4.141
R	Fusiform gyrus (2)	36 -68 -12	16	4.385
L	Inferior temporal/occipital gyrus	-48 -68 -10	182	5.700
L	Posterior intraparietal sulcus	-24 -68 34	60	4.550
L	Superior parietal lobule	24 66 56	29	4.022
R	Superior parietal lobule	24 -64 48	46	4.750
L	Anterior intrapartietal sulcus	-46 -38 40	129	5.066
L	Middle frontal gyrus/ precentral gyrus (FEF)	-28 -4 58	177	4.886
L	Inferior frontal gyrus/precentral gyrus	-40 2 34	215	6.187
L	Dorsomedial PFC	-8 14 50	212	6.177
L	Anterior Insula	-38 20 -4	81	5.038
R	Middle cingulate cortex	8 20 46	14	4.609
L	Anterior cingulate cortex	-6 36 30	14	4.647
L	Inferior frontal gyrus	-48 46 0	17	3.740
R	Cerebellum (lobule VIIb)	30 -70 -50	25	4.193
R	Cerebellum (lobule VI)	34 -62 -28	43	4.744
L	Putamen	-18 10 2	39	5.170
L	Caudate	-14 14 8	45	4.781
Controllable > Uncontrollable				
R	PCC	12 -56 18	49	-4.802

Activation clusters defined based on voxel-level 0.001 threshold, minimum 13 voxel extent: Cluster corrected 0.05. Abbreviations: L, left; R, right, FEF, frontal eye field.

Supplementary Table 2: Exploratory analysis

Hemisphere	Area	MNI coordinates	Cluster extent (voxels)	t-value at peak coordinate
Uncontrollable > Controllable				
R	Angular gyrus	44 -74 34	25	-3.721
L	Supramarginal gyrus	-44 -36 26	17	-4.304
R	Transverse temporal gyrus	44 -24 16	16	-3.637
L	Parietal operculum	-40 -18 20	12	-3.396
R	Superior temporal gyrus	64 -20 -6	24	-4.707
R	Medial frontal gyrus	18 30 44	12	-3.943
L	Medial frontal gyrus	-6 56 26	30	-4.330
L	Putamen	-28 -4 -10	10	-3.942

Activation clusters defined based on voxel-level 0.005 threshold, minimum 10 voxel extent. Note that for a voxelwise threshold of 0.005, a cluster extent of 27 or more voxels is corrected at the cluster level of 0.05. Only Controllable > Uncontrollable reported. A cluster in the posterior cingulate cortex was not reported here as it was included in Supplementary Table 1. Abbreviations: L, left; R, right

Supplementary references

1. Spielberger, C. D., Gorsuch, R. L. & Lushene, R. E. *Manual for the State-Trait Anxiety Inventory*. (Consulting Psychologists Press, 1970).

2. Charpentier, C. *et al.* How representative are neuroimaging samples? Large-scale evidence for trait anxiety differences between MRI and behaviour-only research participants. *ResearchGate* (2020) doi:10.31234/osf.io/cqdne.

3. Feinberg, D. A. *et al.* Multiplexed Echo Planar Imaging for Sub-Second Whole Brain FMRI and Fast Diffusion Imaging. *PLOS ONE* **5**, e15710 (2010).

4. Meyer, C., Padmala, S. & Pessoa, L. Dynamic Threat Processing. *Journal of Cognitive Neuroscience* **31**, 522–542 (2018).

5. Smith, J. F., Hur, J., Kaplan, C. M. & Shackman, A. J. The Impact of Spatial Normalization for Functional Magnetic Resonance Imaging Data Analyses Revisited. *bioRxiv* 272302 (2018) doi:10.1101/272302.

6. Cox, R. W. AFNI: Software for Analysis and Visualization of Functional Magnetic Resonance Neuroimages. *Computers and Biomedical Research* **29**, 162–173 (1996).

7. Pruim, R. H. R., Mennes, M., Buitelaar, J. K. & Beckmann, C. F. Evaluation of ICA-AROMA and alternative strategies for motion artifact removal in resting state fMRI. *NeuroImage* **112**, 278–287 (2015).

8. Avants, B., Tustison, N. & Song, G. Advanced Normalization Tools: V1.0. *The Insight Journal* 681 (2009).

9. Iglesias, J. E., Liu, C.-Y., Thompson, P. M. & Tu, Z. Robust Brain Extraction Across Datasets and Comparison With Publicly Available Methods. *IEEE Transactions on Medical Imaging* **30**, 1617–1634 (2011).

10. Shattuck, D. W. & Leahy, R. M. BrainSuite: an automated cortical surface identification tool. *Med Image Anal* **6**, 129–142 (2002).

11. Greve, D. N. & Fischl, B. Accurate and robust brain image alignment using boundary-based registration. *NeuroImage* **48**, 63–72 (2009).

12. Eklund, A., Knutsson, H. & Nichols, T. E. Cluster failure revisited: Impact of first level design and physiological noise on cluster false positive rates. *Human Brain Mapping* **40**, 1–16 (2001).

13. Cox, R. W. Equitable Thresholding and Clustering. *bioRxiv* 295931 (2018).

14. Bach, D. R., Flandin, G., Friston, K. J. & Dolan, R. J. Time-series analysis for rapid event-related skin conductance responses. *Journal of Neuroscience Methods* **184**, 224–234 (2009).

15. Engelmann, J. B., Meyer, F., Fehr, E. & Ruff, C. C. Anticipatory Anxiety Disrupts Neural Valuation during Risky Choice. *J. Neurosci.* **35**, 3085–3099 (2015).

16. Lim, C. L. *et al.* Decomposing skin conductance into tonic and phasic components. *International Journal of Psychophysiology* **25**, 97–109 (1997).

17. Mumford, J. A., Poline, J.-B. & Poldrack, R. A. Orthogonalization of Regressors in fMRI Models. *PLOS ONE* **10**, e0126255 (2015).

18. McElreath, R. *Statistical Rethinking | Richard McElreath*. (CRC Press, 2020).

19. Lewandowski, D., Kurowicka, D., & Joe, H. Generating random correlation matrices based on vines and extended onion method. *Journal of Multivariate Analysis* **100**, 1989-2001 (2009).
