# Supplementary material for: Controllability over stressor decreases responses in key threat-related brain areas
Source: Commun Biol. 2021 Jan 5;4:42. doi: 10.1038/s42003-020-01537-5 (PMC7785729; doi:10.1038/s42003-020-01537-5)
Supplement: Supplementary file 3 — Description of Additional Supplementary Files [file 42003_2020_1537_MOESM3_ESM.pdf]

Description of Additional Supplementary Files

File Name: Supplementary Video 1
Description: Moving-circles paradigm recently developed where two circles move around the screen, sometimes moving closer and at times moving away from each other. When the circles touch, participants are delivered a mild electric stressor. Note that circle movement, while smooth, has a high degree of unpredictability.
